# An Epidemiological Study on *Salmonella* in Tibetan Yaks from the Qinghai–Tibet Plateau Area in China

**DOI:** 10.3390/ani14243697

**Published:** 2024-12-21

**Authors:** Dengyu Li, Kaiqin Zhang, Xiaofeng Xue, Zhanchun Bai, La Yang, Jingjing Qi, Sizhu Suolang

**Affiliations:** 1 College of Animal Science, Tibet Agricultural and Animal Husbandry University, Nyingchi 860000, China; 202100201101@stu.xza.cdu.cn (D.L.);; 2 Shanghai Veterinary Research Institute, Chinese Academy of Agricultural Sciences China, Shanghai 200241, China; 3 “Fourteenth Five-Year Plan” China Agricultural Rural Ministry Key Laboratory (Jointly Built by the Ministry and Provincial Government), Nyingchi 860000, China

**Keywords:** yaks, drug resistance, multi-locus sequence typing, *Salmonella*, serotype

## Abstract

*Salmonella* is an important foodborne pathogen that can cause a range of illnesses in humans; it has also been a key focus for monitoring in the field of public health, including gastroenteritis, sepsis, and arthritis, and can also cause a decline in egg production in poultry and diarrhea and abortion in livestock, leading to death in severe cases, resulting in huge economic losses. This study aimed to investigate the isolation rate, antimicrobial resistance, serotypes, and genetic diversity of *Salmonella* isolated from yak feces in various regions on the Qinghai–Tibet Plateau. A total of 1222 samples of yak dung were collected from major cities in the Qinghai–Tibet Plateau area, and the sensitivity of the isolated bacteria to 10 major classes of antibiotics was determined using the K-B paper disk diffusion method for drug susceptibility. Meanwhile, the serotypes of the isolated bacteria were analyzed using the plate agglutination test for serum antigens, and their carriage of drug resistance and virulence genes was determined using PCR and gel electrophoresis experiments. The isolated bacteria were also classified using MLST (Multi-Locus Sequence Typing). The overall isolation rate for *Salmonella* was 18.25% (223/1222), and the results of the antibiotic susceptibility tests showed that 98.65% (220/223) of the isolated bacteria were resistant to multiple antibiotics. In the 223 isolates of *Salmonella*, eight classes of 20 different resistance genes, 30 serotypes, and 15 different types of virulence genes were detected. The MLST analysis identified 45 distinct sequence types (STs), including five clonal complexes, of which ST34, ST11, and ST19 were the most common. These findings contribute valuable information about strain resources, genetic profiles, and typing data for *Salmonella* in the Qinghai–Tibet Plateau area, facilitating improved bacterial surveillance, identification, and control in yak populations. They also provide certain data supplements for animal *Salmonella* infections globally, filling research gaps.

## Simple Summary

As a major economic pillar in Tibet, the yak breeding industry is an important source of income and livelihood for local farmers and herdsmen. *Salmonella*, one of the most important foodborne pathogens globally, has seriously threatened the healthy development of this industry for many years, causing huge economic losses for local herdsmen and related industries. Furthermore, there are many serotypes of *Salmonella* that can infect different hosts, causing varying degrees of damage. The aim of this study was to investigate the prevalence of *Salmonella* in yak farms in the Qinghai–Tibet Plateau area. A total of 223 strains of *Salmonella* were isolated from 1222 fecal samples from yaks from Tibet and Qinghai, with an overall isolation rate of 18.25% (223/1222). These 223 *Salmonella* isolates were serotyped, and their antimicrobial susceptibility, resistance genes, and virulence genes were investigated. Furthermore, MLST (Multi-Locus Sequence Typing) was applied to the isolates. The results showed that the carriage of *Salmonella* in Tibetan yaks is quite serious in the Qinghai–Tibet Plateau area, with Enteritidis *Salmonella* (17.94%) and Typhimurium *Salmonella* (13.90%) representing the predominant serotypes. Given that the *Salmonella* isolates also showed resistance to antimicrobial drugs, continuous monitoring of *Salmonella* infections and resistance in the Qinghai–Tibet Plateau area is needed to avoid the potential risk of foodborne disease transmission.

## 1. Introduction

Infectious diseases of bacterial origin widely affect humans and animals throughout the world [1,2]. *Salmonella* is a Gram-negative facultative anaerobic intracellular pathogen that ranges in size from 2 to 3 µm and belongs to the *Enterobacteriaceae* family. It is divided into two species: enteric *Salmonella* and Bongor *Salmonella*. It is further divided into typhoid and non-typhoid categories, and over 2600 serotypes have been identified [3,4], with intestinal *Salmonella* causing a significant burden, accounting for more than 180 million cases of diarrhea annually (9% of cases globally) [5]. *Salmonella* is the second most frequently reported foodborne gastrointestinal infection in the European Union and also the main cause of foodborne outbreaks in EU member states and non-member states. The number of deaths from outbreaks was the highest ever reported in the EU in the last 10 years, mainly caused by L. monocytogenes and to a lesser degree by *Salmonella*. *Salmonella* and in particular *S. Enteritidis* remained the most frequently reported causative agent for foodborne outbreaks [6]. Biao Tang et al. isolated *Salmonella* strains from five provinces in China (Zhejiang, Guangdong, Jiangxi, Hunan, and Qinghai) and reported that pigs, chickens, ducks, and geese—major food-source animals—were reservoirs of the pathogen [7]. As reported by UN regions, 73 studies from 27 countries in Africa showed that the prevalence of *Salmonella* in poultry samples was 13.9% [8]. In South Asia, the prevalence of *Salmonella* isolated from human, animal, and environmental samples was as high as 14.47% [9]. In the United States from 2012 to 2019, 27 outbreaks of *Salmonella* were linked to beef consumption, resulting in 1103 illnesses, 254 hospitalizations, and two deaths. The US Department of Agriculture (USDA) Economic Research Service estimates that the average total cost of NTS-related diseases in the United States, including medical expenses and productivity losses, is USD 4.1 billion (USDA Economic Research Service) [10]. The disease is prevalent in cattle, particularly during the summer grazing period, and affects both adults (sporadically) and calves (epidemically), impacting herd health and posing a risk to human health through contaminated animal products and excrement. Yak is a unique breed of cattle that lives at an altitude of 3000–5000 m in the Qinghai–Tibet Plateau in China, with a population of about 13.5 million heads. It is a native species and an important source of milk, meat, wool, hide, and dung for the local economy and the livelihoods of herders. It is also exported for high-end foreign consumption [11,12]. Cow diarrhea causes huge economic losses for herders; a high proportion is caused by *Salmonella*, which is common in cows and significantly harms the cattle industry [13]. When *Salmonella* infects cows, it causes sepsis, gastroenteritis, and localized tissue inflammation [14]. It penetrates the intestinal mucosa [15] and changes the structure of the cytoskeleton of intestinal cells, causing the microvilli to collapse and reducing the intestinal epithelium’s ability to absorb water and nutrients [16].

Previous use of chloramphenicol and Co-trimoxazole led to the emergence of MDR (multidrug-resistant) typhoid *Salmonella* strains [17,18]. Meanwhile, in recent decades, the misuse of antimicrobials has also led to a rapid increase in the number of *Salmonella* strains that are resistant to ciprofloxacin (CIPR) [19]. Multidrug-resistant *Salmonella* strains have been isolated from duck samples in Vietnam [20], cow samples in Nigeria [21], pig samples in South Korea [22], chicken samples in China [23], and animal product samples in South Africa [24], indicating that multidrug resistance in *Salmonella* is a serious problem worldwide. However, given that little is known about the prevalence and antimicrobial resistance of *Salmonella* in yaks, studying the antimicrobial susceptibility of *Salmonella* in yak products in China is crucial to effectively controlling its spread.

With the development of molecular biology and genomics, many molecular typing techniques have been employed to assess bacterial diversity [25]. Among these, Multi-Locus Sequence Typing (MLST) analysis is a useful molecular analysis tool that uses allelic differences in housekeeping gene sequences to distinguish strains [26]. This biological method is commonly used to characterize bacteria and is considered a gold-standard method for bacterial typing [27]. The method is relatively simple. It involves the PCR amplification of housekeeping gene sequences, followed by sequencing and a database query to determine the sequence types [28]. MLST has increasingly been used for strain comparison and has become an important method in bacterial molecular epidemiology research.

However, existing research on the prevalence, antimicrobial resistance, and molecular typing of *Salmonella* in yak dung on the Qinghai–Tibet Plateau has been very limited so far. This study conducted a preliminary investigation of the infection status of *Salmonella* bacteria in the main livestock areas of the Tibetan Plateau. Experiments were conducted to determine the sensitivity of the isolated and identified bacterial strains to major antimicrobials, and serotype analysis, an investigation of major drug resistance and virulence genes, and MLST typing were carried out. This study not only provides data on the incidence of *Salmonella* in yak in the Qinghai–Tibetan Plateau area, but also provides data for monitoring the global food safety of biological products as the import and export volume of yak and its by-products increases. However, this study did not conduct an epidemiological investigation of *Salmonella* in all regions of the Tibetan Plateau, and a random error occurred during sample selection, so the results of this experiment have certain limitations and can only provide certain reference data for the prevention and control of *Salmonella* bacterial disease and related investigations.

## 2. Materials and Methods

### 2.1. Sample Collection

**Figure 1:**
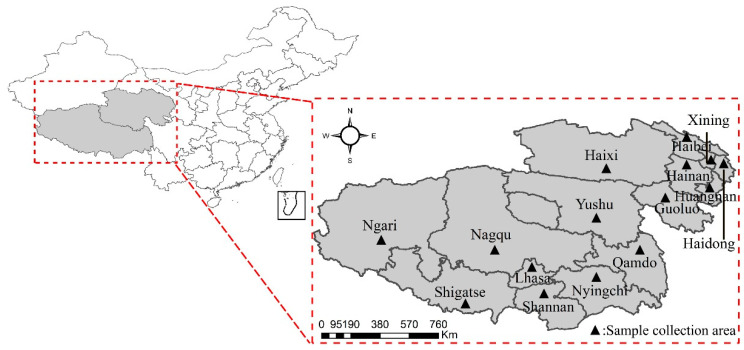
Geographical distribution of sample collection sites (marked with ▲).

During the period from September 2019 to September 2022, 1222 yak fecal samples (10–20 g) were collected in the primary yak breeding regions of the Qinghai–Tibet Plateau, China. In each prefecture-level city, the researchers selected samples of yaks of different ages and sexes at random based on local breeding conditions, effectively avoiding data distortion caused by human bias. As shown in Figure 1, these carefully selected samples were collected from 15 different locations across the city, including but not limited to Lhasa, Shigatse, and Shannan, which are important breeding areas on the Tibetan Plateau. These places are not only famous pastoral areas on the Tibetan Plateau but also intersections of various ecological environments, providing diverse sources of data. The minimum sample size is determined by the formula described by Thrusfield [29], with a confidence level of 99% and precision of 5%, considering a 20% estimated prevalence rate, which leads to the minimum number of samples required being approximately 1000. In each city, the three-point sampling method is adopted, where at least three different locations of yak farming areas are selected for sampling. The method used for sample collection was random simple sampling without differentiation between the age and gender of the yaks, ensuring an equal chance of selecting yaks from each breeding farm. Rectal swabbing was used in each selected yak: a sterile swab was moistened with physiological saline, inserted into the rectum to about 6–7 cm, and gently rotated to remove a small amount of feces, which was then placed in a sterile fecal box, sealed, and stored at 4 °C in a refrigerator before its transportation to the Center for High-Altitude Animal Infectious Diseases at the Tibetan Agricultural and Animal Husbandry University for further analysis. The specific information and source of the yak dung samples collected, as well as the condition of the yak, are provided in Supplementary Material Data Sheet S2.

### 2.2. Isolation and Cultivation of Salmonella

*Salmonella* was isolated and cultivated following the established protocols in [30,31]. The fecal samples were first enriched using Buffered Peptone Water (BPW, Becton, Dickinson and Company, Franklin Lakes, NJ, USA) at 42 °C with constant agitation at 230 rpm for 24 h. The enrichment broth was further screened on an improved semi-solid Rappaport–Vassiliadis (MRSV, Becton, Dickinson and Company, Franklin Lakes, NJ, USA) culture medium and incubated overnight at 42 °C. The enriched bacteria were screened on MRSV semi-solid, SS agar, and BS agar (Hangzhou Microbial Reagent Co., Ltd, Hangzhou, China) selective media based on their morphology, color, Gram staining, and growth characteristics.

Further confirmation of the suspected *Salmonella* strains was achieved via *InvA* detection, and the primer sequences used in this test are listed in Supplementary Table S2. The strains that showed a specific band during the gel electrophoresis experiment were considered positive for *Salmonella*. These positive isolates underwent drug resistance and virulence gene detection, antimicrobial susceptibility testing, MLST (Multi-Locus Sequence Typing), and phylogenetic analysis.

### 2.3. Antimicrobial Susceptibility Testing

The antimicrobial susceptibility of the isolates was assessed using the Kirby–Bauer disk diffusion method, as per the CLSI guidelines [32]. Single colonies were inoculated into TSB (Hangzhou Microbial Reagent Co., Ltd, Hangzhou, China) liquid culture medium and incubated at 41 °C in a constant-temperature incubator. The concentration of the bacterial suspension was determined at 600 nm using a McFarland turbidimeter, with the D_600nm_ value set to 0.6–0.8. The appropriate absorbance was calculated from the results of a preliminary experiment, which showed that the bacterial concentration was approximately 1.5 × 10^8^ CFU/mL at this value, consistent with the 0.5 McFarland standard required. Then, 300 μL of the bacterial supernatant was evenly spread onto Mueller–Hinton Agar (MHA) (Solaibao Technology Co., Ltd., Beijing, China). A total of 26 antimicrobials (Hangzhou Microbial Reagent Co., Ltd, Hangzhou, China) commonly used in both veterinary and human medicine were employed: the beta-lactam antimicrobials cefotaxime (30 µg), cefazolin (30 µg), ampicillin (10 µg), cefuroxime (30 µg), cefoperazone (30 µg), penicillin (10 UI), cephalexin (30 µg), and oxacillin (10 µg); the macrolide antimicrobials azithromycin (30 µg) and erythromycin (15 µg); the aminoglycoside antimicrobials gentamicin (10 µg), kanamycin (30 µg), and streptomycin (10 µg); the tetracycline antimicrobials minocycline (30 µg), doxycycline (30 µg), and tetracycline (30 µg); the lincosamide antimicrobial lincomycin (2 µg); the chloramphenicol antimicrobials chloramphenicol (30 µg) and florfenicol (30 µg); the polypeptide antimicrobials vancomycin (30 µg), bacitracin (10 UI), and polymyxin B (300 µg); the quinolone antimicrobial ciprofloxacin (5 µg); nalidixic acid (5 µg); the nitrofuran antimicrobial furazolidone (300 µg); and the sulfonamide antimicrobial sulfamethoxazole (300 µg). Antimicrobial disks were placed in triplicate on the agar surface, and the criteria for resistance outlined in Supplementary Table S1 were followed. The inhibition zone diameters were measured, and resistance was determined in accordance with the CLSI standards and the manufacturer’s instructions (Hangzhou Microbial Reagent Co., Ltd, Hangzhou, China). The quality control strain used in this experiment was *E. coli* ATCC25922 (China center of industrial culture collection, Beijing, China), which is a standard strain. Differences in the drug susceptibility of the *Salmonella* strains from different regions were analyzed using a one-way ANOVA. In order to ensure the rigor of the experiments, positive and negative controls were used in the PCR experiments, with *Salmonella* ATCC35640 (China center of industrial culture collection, Beijing, China) as the positive control and ddH_2_O as the negative control.

### 2.4. DNA Extraction

To extract bacterial DNA from the *Salmonella* isolates, a single colony was first purified and inoculated into TSB liquid medium at 37 °C until the optical density at 600 nm (OD_600nm_) reached 0.6–0.8. Then, DNA was extracted from the bacteria using a JetFlex Genomic DNA Purification Kit (Thermo Fisher Scientific—CN, Shanghai, China) and stored at −20 °C, with all operations strictly following the JetFlex™ Genomic DNA Purification Kit User Guide.

### 2.5. Detection of Antimicrobial Resistance Genes and Virulence Genes

Primers were designed in accordance with previously published research [33]. PCR was used to detect the common intestinal toxin *STN*, pathogenicity island-1 (*orgA*, *sopE*, *avrA)*, pathogenicity island-2 (*ttrB*, *sseL*), pathogenicity island-3 (*rhuM*, *mgtC*), pathogenicity island-4 (*orfL*), pathogenicity island-5 (*sopB*, *pipD*), the oxidative stress gene *sodC1*, the adhesion gene *lpfC*, the toxin plasmid virulence genes *spvC* and *pefA*, as well as the specific *invA* genes and genes encoding resistance to 8 classes of antimicrobials, including β-lactams, tetracyclines, quinolones, sulfonamides, chloramphenicol, macrolides, peptides, and lincomycin (Supplementary Table S2). Each PCR amplification was carried out with a total volume of 50 μL of a mixture containing 1 μL of each primer, 2 μL of the genomic DNA template (50 ng/μL), 25 μL of 2× Taq DNA polymerase (Thermo Fisher Scientific—CN, Shanghai, China), and 21 μL of ddH_2_O. The amplified products were analyzed using electrophoresis on 1.5% agarose gel, followed by gel documentation (Bio-Rad, Hercules, CA, USA). Purification was performed using the GeneJET Gel Extraction Kit (Thermo Fisher Scientific—CN, Shanghai, China).

### 2.6. Statistical Analysis of Data

The sequences of the resistance genes and virulence genes detected in the isolated strains were compared with the corresponding sequences on the NCBI website (https://www.ncbi.nlm.nih.gov/) to confirm the results. Then, a one-way ANOVA was conducted to investigate the distribution of resistance, resistance genes, and virulence genes among the strains according to the different regions of isolation.

### 2.7. Salmonella Serotype Identification

According to Reference [34], all the isolates were serotyped via slide agglutination using commercial antisera (Thermo Fisher Scientific—CN, Shanghai, China). First, the isolates were cultured overnight on Columbia blood agar and Swarm agar (Becton, Dickinson and Company, Franklin Lakes, NJ, USA). One bacterial colony was taken from the Columbia blood agar for the agar gel immunodiffusion test. A total of 20 μL of OMA, one of the four O-group monovalent antisera, was used. OMA, OMB, OMC, and OMD represent four different subtypes of the O antigen of *Salmonella*, which are prepared for different subtypes of the *Salmonella* O antigen. Visible agglutination was considered a positive result indicating that the O antigen in the isolate belonged to the O group. Then, the agglutination results for H antigens were verified using each of the O-antigen-specific antisera from the previously confirmed O-multiantigen serum to verify the specificity of the H antigen in the isolated material. A bacterial population was taken from the Swarm agar for the slide agglutination test, using 20 μL of polyvalent antiserum for each of 4 H groups: HMA; H:E; H:G; and HMC. On the basis of previously established results, a hemagglutination test was performed using each of the H-antigen-specific sera from the H-specific polyvalent antisera of the H group to verify the specificity of the H antigen in the isolate. When no second-phase H antigen was detected in the two-phase isolate according to hemagglutination, the phase was reduced according to the instructions for the commercial antisera. Physiological saline was used as a negative control in each hemagglutination test. Serotypes were determined according to the Kauffmann–White scheme [35] based on the single O and H antigens. A motility test was performed on suspected non-motile isolates. A sterile needle was used to pick colonies from the Columbia blood agar plate and inoculate them into tubular semi-solid culture medium (nutrient agar at a 0.5% concentration) (Hangzhou Microbial Reagent Co., Ltd., Hangzhou, China). When an inoculation line was clear and did not spread, the isolate was considered non-motile. On the other hand, when an inoculation line was unclear and the isolate showed diffused growth, it was considered motile.

### 2.8. Master Gene Profiling with Multi-Locus Sequence Typing

PCR amplification of the housekeeping genes, including *aroC*, *dnaN*, *hemD*, *hisD*, *purE*, *sucA*, and *thrA*, was performed following the protocol outlined in S. et al.’s report [36]. The DNA base sequence is described in Supplementary Table S2.

The PCR products were initially separated on 1.5% agarose gel, followed by the use of an agarose gel DNA recovery kit (TaKaRa, Tokyo, Japan) and DNA concentration/purity assessments using a nucleic acid/protein detector. The purified DNA fragments were then ligated into the pMDTM18-T vector, incubated overnight at 4 °C, and transformed into DH5α competent cells (TaKaRa, Tokyo, Japan) for selection on LB agar containing Amp+. For positive bacterial solutions, plasmid isolation was performed using a High Pure Plasmid Isolation Kit (TIANGEN, Beijing, China), and sequencing was outsourced to Qingdao Biotech Co., Ltd. The MLST analysis, based on the scheme available at https://pubmlst.org/, involved processing the isolates through a standardized protocol [37,38]. After treating the bacteria on TSA and incubating them at 37 °C for 18–24 h, single colonies were selected, lysed using a protocol involving proteinase K (TIANGEN, Beijing, China), and heated at 80 °C for 10 min. The suspension was then maintained at 55 °C for 10 min to lyse the cells, and the DNA was centrifuged, diluted, and stored at −20 °C for future use.

To perform MLST, PCR amplification was conducted for seven specific genes (*aroC*, *dnaN*, *hemD*, *hisD*, *purE*, *sucA*, and *thrA)* using 50 μL of a reaction mixture containing primers, Taq polymerase, the template DNA, and water. The thermal cycling conditions for the PCR reaction were as follows: initial denaturation at 94 °C for 30 s, followed by 30 cycles of denaturation at 95 °C for 30 s, annealing at 55 °C for 30 s, extension at 76 °C for 30 s, and a final extension at 75 °C for 2 min, after which the conditions were held at 4 °C. The PCR products were loaded onto 1% agarose gel, run at 100 V for 60 min, and visualized using an Alpha Innotech imaging system(FluorChem FC3).

### 2.9. Multi-Locus Sequence Typing and Phylogenetic Analysis

Seven housekeeping genes (*thrA*, *purE*, *sucA*, *hisD*, *aroC*, *hemD*, and *dnaN*) from the 223 *Salmonella* isolates were sequenced, followed by alignment, assembly, and trimming [39]. BioNumerics 8.1 (Applied Maths, Keistraat, De Pinte, Belgium) was employed to assign allele numbers to the processed gene sequences, enabling the assignment of a sequence type (ST) to each strain. A dendrogram was generated using the allele numbers from the seven loci, with similarity coefficients calculated through clustering methods, specifically, the construction of a minimum spanning tree (MST). Strains were classified as clonal complexes (CCs) if they exhibited identical alleles in four or more loci. The clustering analysis employed the unweighted pair group method with arithmetic mean (UPGMA) for evolutionary genetic studies. To assess the polymorphism of the allele loci and the strain diversity, Simpson’s diversity index (Ds) was quantitatively analyzed, differentiating the strains from various regions. The formula for Simpson’s diversity index is D = 1 − Σ(Ni/N)^2^, where D indicates the diversity index, N represents the total number of individuals of all species, Ni represents the number of individuals of the i-th species, and S represents the number of species. The software used to construct the minimum spanning tree and the phylogenetic tree included CloudCompare (v2.11.3), GrapeTree (v1.20), and Mega (v7.0).

## 3. Results

### 3.1. The Prevalence of Salmonella

The results showed that 223 out of 1222 samples (18.25%) tested positive for *Salmonella*. Table 1 shows the specific isolation rates for *Salmonella*, with 127 out of 585 samples (21.71%) testing positive in Tibet and 96 out of 637 samples (15.07%) testing positive in Qinghai province. The specific information of the 223 fecal samples is listed in Data Sheet 2.

### 3.2. Results of the Antimicrobial Resistance Tests

**Figure 2:**
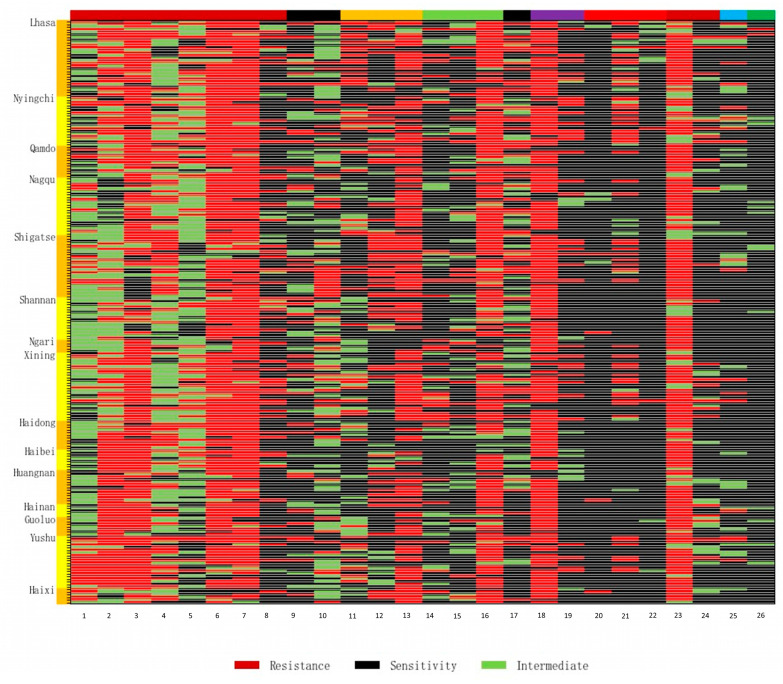
Antimicrobial resistance heatmap of 223 *Salmonella* isolates from the Tibetan Plateau. The regions from which samples were sourced are on the left vertical axis, and the antimicrobials (1–26) are on the horizontal axis. The color scale bar represents the diameter of the inhibition zone, ranging from 0 to 40 mm. (1) Cefotaxime; (2) cefazolin; (3) ampicillin; (4) cefuroxime; (5) cefoperazone; (6) penicillin; (7) cephalexin; (8) oxacillin; (9) azithromycin; (10) erythromycin; (11) gentamicin; (12) kanamycin; (13) streptomycin; (14) minocycline; (15) doxycycline; (16) tetracycline; (17) lincomycin; (18) chloramphenicol; (19) florfenicol; (20) vancomycin; (21) bacitracin; (22) polymyxin B; (23) ciprofloxacin; (24) nalidixic acid; (25) furazolidone; and (26) sulfamethoxazole.

**Figure 3:**
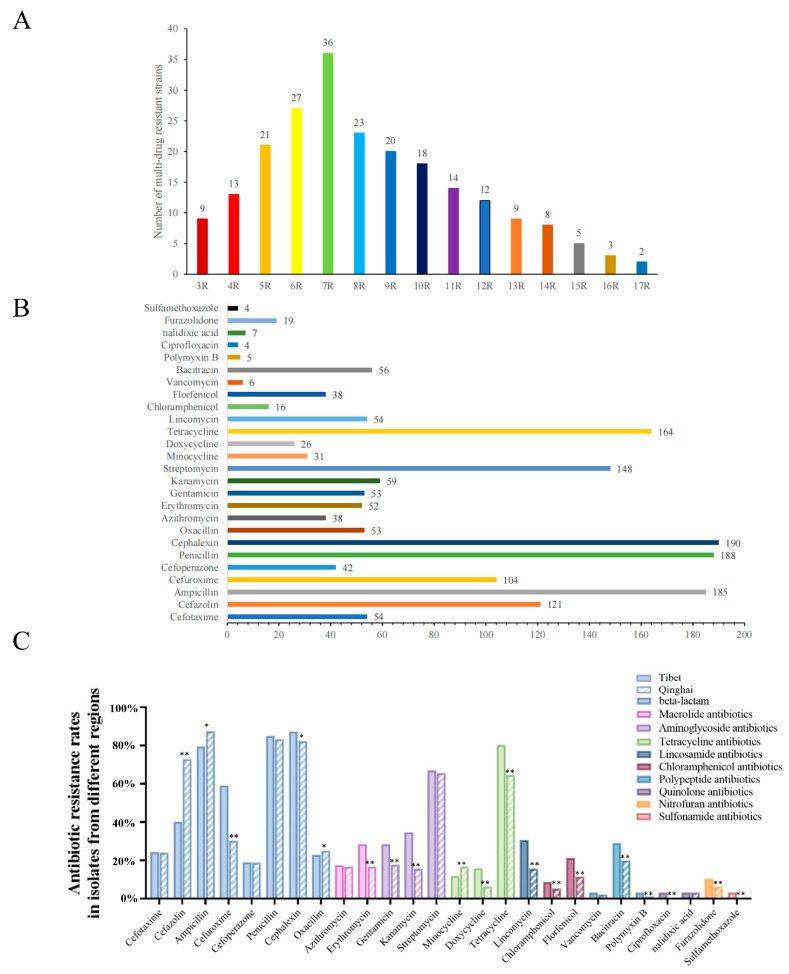
Specific antimicrobial resistance of *Salmonella* on the Qinghai–Tibet Plateau. (**A**) Multidrug resistance of 223 strains of *Salmonella* from yaks on the Qinghai–Tibet Plateau. (**B**) The specific number of drug-resistant *Salmonella* isolates from yaks on the Qinghai–Tibet Plateau. (**C**) The antimicrobial resistance differences in *Salmonella* isolates from Tibet and Qinghai. The bar chart shows the percentage of resistant strains. * indicates a significant difference (*p* < 0.05). ** indicates a highly significant difference (*p* < 0.01).

The strains isolated in this study showed broad-spectrum antimicrobial resistance. After observing the differences in drug resistance across various regions through a resistance heat map (Figure 2), the highest resistance was found for cephalexin (85.20%, 190/223), followed by penicillin (84.30%, 188/223), ampicillin (82.96%, 185/223), tetracycline (73.54%, 164/223), streptomycin (66.37%, 148/223), cefazolin (54.26%, 121/223), and cefuroxime (46.64%, 104/223). The majority of the isolated strains were sensitive to ciprofloxacin (1.79%, 4/223), sulfamethoxazole (1.79%, 4/223), cefotaxime (18.83%, 42/223), polymyxin B (2.24%, 5/223), vancomycin (2.69%, 6/223), and nalidixic acid (3.59%, 8/223) (Figure 3A).

The resistance rates of the *Salmonella* isolates to the antimicrobials varied across different regions (Figure 3C). The *Salmonella* isolates from the Tibet and Qinghai regions showed resistance rates to β-lactam antimicrobials (cephalothin, penicillin, ampicillin, and cefazolin), aminoglycosides (streptomycin), and tetracyclines (Tetracycline) exceeding 60%. In particular, the resistance rate against the β-lactam antimicrobial cephalothin is the highest in the Tibet region (87.74%), while the resistance rate against the β-lactam antimicrobial ampicillin is the highest in the Qinghai region (87.50%). In both regions, there is relatively high resistance to erythromycin (Tibet: 28.35%; Qinghai: 16.67%), kanamycin (Tibet: 34.65%; Qinghai: 15.63%), and gentamicin (Tibet: 29.13%; Qinghai: 19.79%). Relatively low resistance to azithromycin (Tibet: 17.32%; Qinghai: 16.67%), minocycline (Tibet: 11.81%; Qinghai: 16.67%), doxycycline (Tibet: 15.75%; Qinghai 6.:25%), and Fluoroquinolones (Tibet: 21.26%; Qinghai: 11.46%) was seen, while there was almost no resistance to ciprofloxacin (Tibet: 3.15%, Qinghai: 0%), nalidixic acid (Tibet: 3.15%; Qinghai: 3.13%), and sulfamethoxazole (Tibet: 3.15%; Qinghai: 0%). Overall, both regions showed strong resistance to first-generation antimicrobials such as cephalothin, penicillin, streptomycin, and tetracycline. This resistance decreased for second-generation antimicrobials such as cefuroxime, benzylpenicillin, minocycline, and doxycycline. The resistance to third-generation antimicrobials like Ceftriaxone and the fourth-generation antimicrobial sulfamethoxazole is lower, and there is almost no resistance to Quinolones.

Among different definitions, multidrug resistance (MDR) is defined as acquired non-susceptibility to at least one agent from three or more antimicrobial categories [40]. The proportion of multidrug-resistant *Salmonella* strains in our study was 98.65% (220/223), with 29.17% (42/223) of the isolated strains showing resistance to four classes of antimicrobials. Among them, 147 strains (65.92%, 147/223) were resistant to five–eight types of antimicrobials, and 2 strains were resistant to nine classes of antimicrobials (Figure 3B).

The resistance of the isolated strains from Tibet to the 10 classes of antimicrobials was generally higher than in Qinghai. The resistance rates of the isolated strains to cefuroxime, erythromycin, gentamicin, kanamycin, doxycycline, tetracycline, lincomycin, florfenicol, bacitracin, and furazolidone are significantly higher in the Tibetan samples than in those from Qinghai. However, the resistance of the Tibetan strains to cefazolin, ampicillin, and minocycline was significantly lower than in the strains from Qinghai. The specific resistance patterns of the strains are shown in Table S3.

### 3.3. Results of PCR-Based Detection of Antimicrobial Resistance Genes in Salmonella

The antimicrobial resistance genes carried by the 223 *Salmonella* isolates from yaks were analyzed using PCR. The results showed that the presence of antimicrobial resistance genes in the 223 *Salmonella* isolates was diverse, with a total of 20 different resistance genes detected for eight classes of antimicrobials.

**Figure 4:**
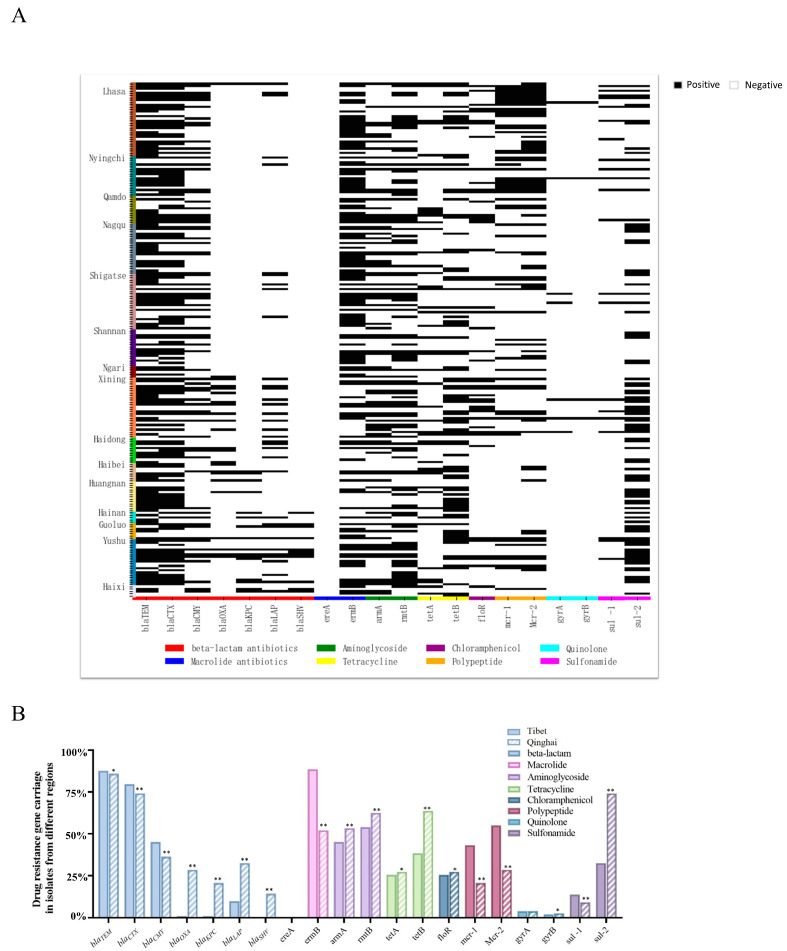
Prevalence of *Salmonella* resistance genes on the Qinghai–Tibet Plateau. (**A**) Antimicrobial resistance gene carriage in 223 *Salmonella* isolates from yaks from the Qinghai–Tibet Plateau region. (**B**) Differences in the carriage of antimicrobial resistance genes in *Salmonella* isolates from Tibet and Qinghai. * indicates a significant difference (*p* < 0.05). ** indicates a highly significant difference (*p* < 0.01).

The top 10 antimicrobial resistance genes detected are *bla_TEM_*_,_ *bla_CTX_*_,_ *ermB*, *rmtB*, *sul-2*, *tetB*, *armA*, *Mcr-2*, *bla_CMY_*, and *mcr-1* (Figure 4A). A proportion of 42.60% of the strains carry aminoglycoside resistance genes, 30.94% carry peptide resistance genes, 30.27% carry tetracycline resistance genes, 29.15% carry macrolides resistance genes, 29.02% carry chloramphenicol resistance genes, 24.89% carry sulfonamide resistance genes, and 2.47% carry quinolone resistance genes.

The results revealed that the prevalence of the *bla_CMY_*, *bla_KPC_*, *bla_SHV_*, *ermB*, *mcr-1*, and *Mcr-2* genes in the isolates from Tibet was higher than in the isolates from Qinghai (Figure 4B). Conversely, the prevalence of the *bla_OXA_*, *bla_LAP_*, *tetB*, and *sul-2* genes was significantly lower in Tibet compared to Qinghai. The macrolide resistance gene (*ereA*) was not found in samples from either region, and the prevalence of the quinolone resistance genes (*gyrA* and *gyrB*) was also the lowest in both areas.

### 3.4. Results of PCR-Based Detection of Virulence Genes in Salmonella

**Figure 5:**
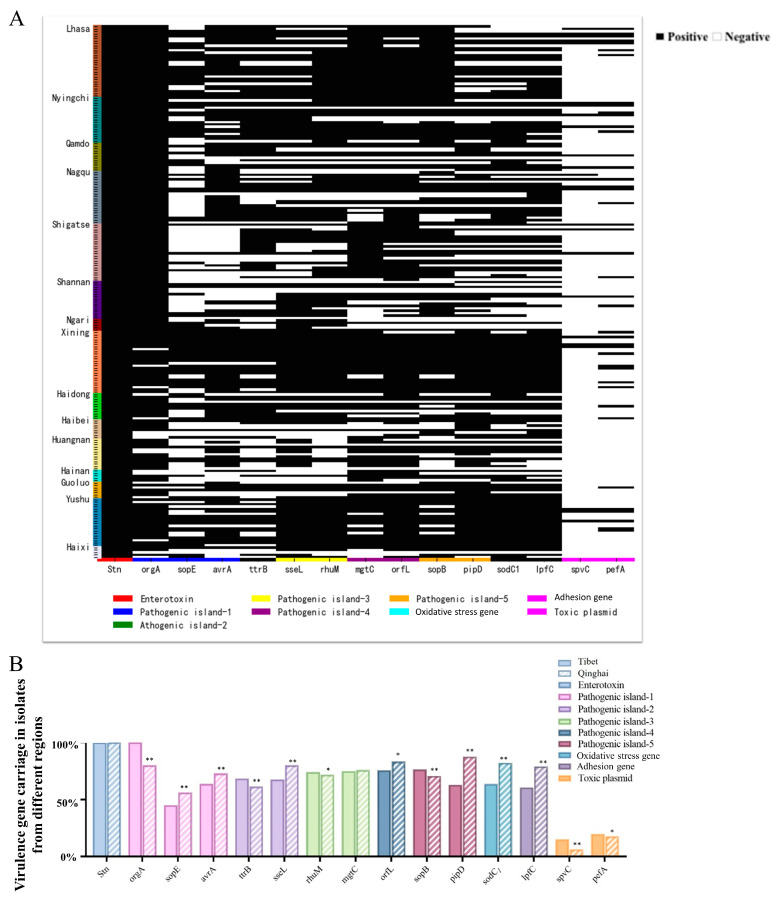
Relationships between *Salmonella* pathogenicity genes in the Qinghai–Tibet Plateau. (**A**) The presence of virulence genes in 223 strains of *Salmonella* isolated from yaks in the Qinghai–Tibet Plateau region. (**B**) Differences in the presence of virulence genes in *Salmonella* isolates from Tibet and Qinghai. * indicates a significant difference (*p* < 0.05). ** indicates a highly significant difference (*p* < 0.01).

PCR testing for virulence genes in the isolated strains revealed that the 223 *Salmonella* isolates carry nine different types of virulence genes (Figure 5A). The highest detection rate, at 100%, was found for the Enterotoxin virulence gene *STN*, followed by those for *orgA* (91.48%), *orfL* (78.92%), mgtC (75.34%), sopB (73.99%), *pipD* (78.92%), *orfL* (73.54%), *sseL* (73.09%), *rhuM* (73.09%), and *sodC1* (71.75%). The detection rates for the *lpfC*, *avrA*, *ttrB*, and *sopE* virulence genes ranged from 70% to 40%, while the detection rates for the remaining virulence genes *pefA* and *spvC* were relatively low (10–20%).

Additionally, there were certain differences in the detection rates of virulence genes based on geographical location (Figure 5B). The detection rates of the virulence genes *orgA*, *ttrB*, *spvC*, and *pefA* were significantly higher in the strains isolated from Tibet than in those from Qinghai. Conversely, the detection rates of the virulence genes *sopE*, *avrA*, *sseL*, *pipD*, *sodC1*, and *lpfC* were significantly lower in Tibet than in Qinghai.

### 3.5. Serotyping of Salmonella Isolated from Yaks on the Qinghai–Tibet Plateau

**Figure 6:**
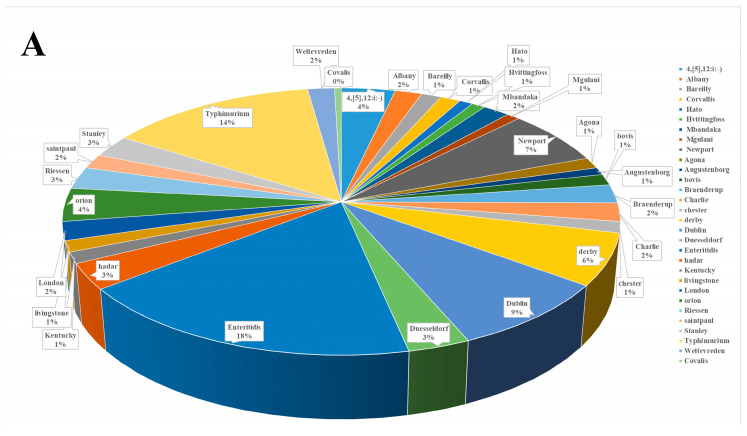

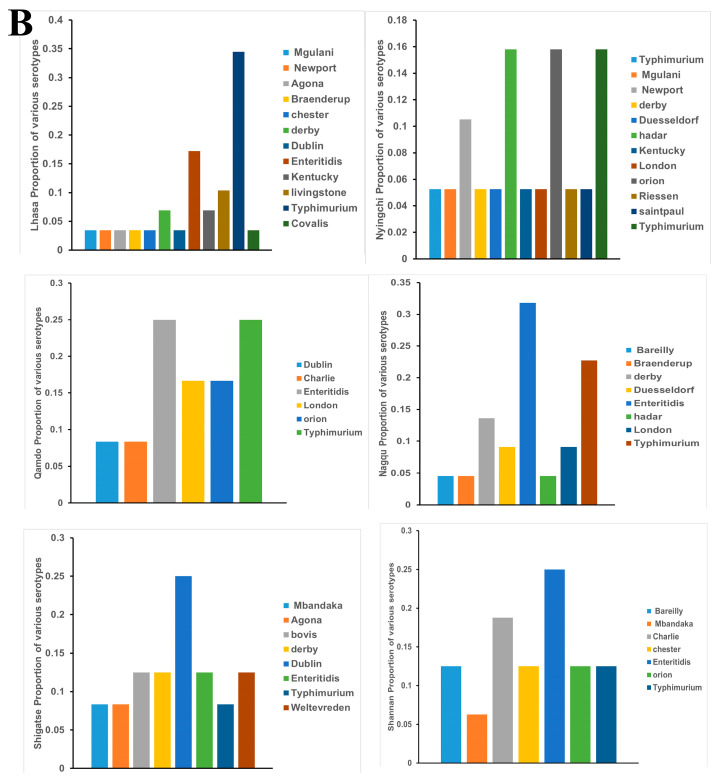

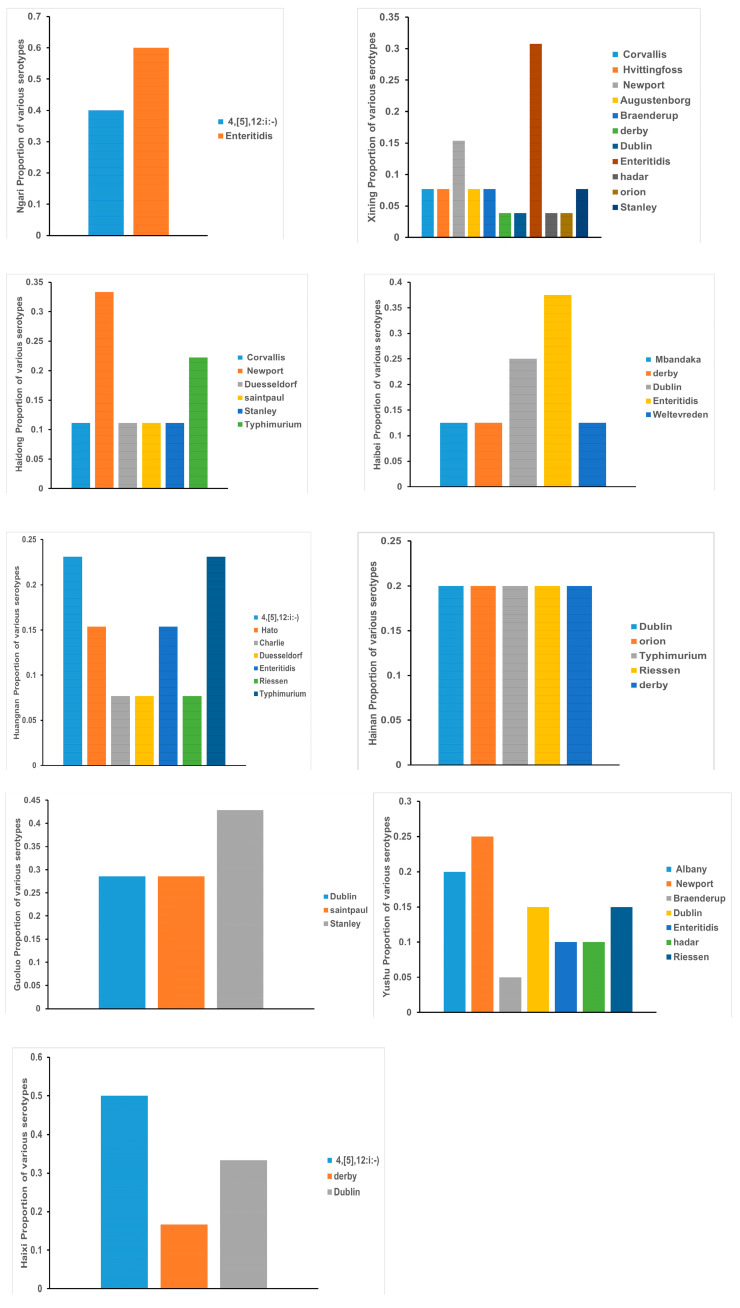
Information on serotypes of *Salmonella* on the Qinghai–Tibet Plateau. (**A**) The distribution of the serotypes of 223 strains of *Salmonella* isolated from yaks in the Qinghai–Tibet Plateau region. (**B**) The distribution of the serotypes of *Salmonella* isolates in different regions of Tibet and Qinghai.

In this study, 223 strains of *Salmonella* were serotyped into 30 different serotypes using glass-plate serum agglutination (Figure 6A). The most common serotype of *Salmonella* isolated from yaks on the Qinghai–Tibet Plateau was Enteritidis (n = 40), followed by Typhimurium (n = 31), Dublin (n = 19), Newport (n = 15), Derby (n = 14), Orion (n = 9), and 4,[5],12: i:-) (n = 8).

Duesseldorf and Hadar each accounted for 3.14% (n = 7). Riessen and Stanley accounted for 2.69% each (n = 6). Braenderup, Charlie, and London each accounted for 2.24% (n = 5). Albany, Mbandaka, Saintpaul, and Weltevreden each accounted for 1.79% (n = 4). Bareilly, Corvallis, Agona, Bovis, Chester, Kentucky, and Livingstone each accounted for 1.35% (n = 3). Hato, Hvittingfoss, Mgulani, and Augustenborg each accounted for 0.90% (n = 2). Covalis was only isolated once.

Based on their different geographical sources, the serotypes of the 223 strains of *Salmonella* isolates were categorized by region (Figure 6B). The most abundant serotypes were found in Lhasa, Tibet, (n = 12) and Xining, Qinghai (n = 11). Enteritidis and Typhimurium were the dominant serotypes in the Tibetan region. Enteritidis was not detected in Linzhi, while Typhimurium was not found in Ngari.

The serotypes Newport and 4,[5],12:i:-) were more prevalent in the Qinghai region, while they were less frequently detected in the Tibetan region. Albany was only detected in Yushu, Qinghai, (n = 4) during this study.

Our findings are consistent with recent reports: *Salmonella* enterica serovars, specifically, the *Salmonella enterica* serovar Typhimurium and the *Salmonella enterica* serovar Heidelberg, are the main serotypes of *Salmonella* found in animal, food, or human sources [41,42].

### 3.6. Salmonella ST and Minimum Spanning Tree Analysis

The *aroC* gene showed the highest polymorphism (n = 32), while the *purE* gene exhibited the lowest polymorphism (n = 15). The average number of alleles across all loci was 10.47 (Table 2).

**Figure 7:**
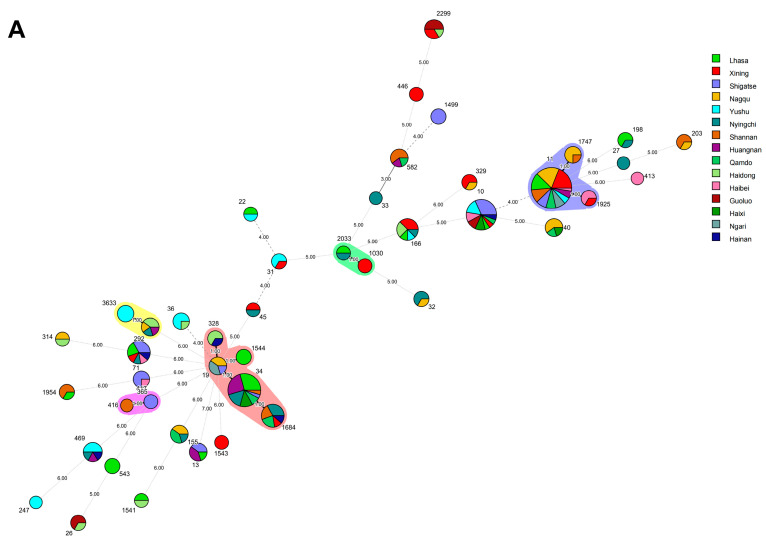

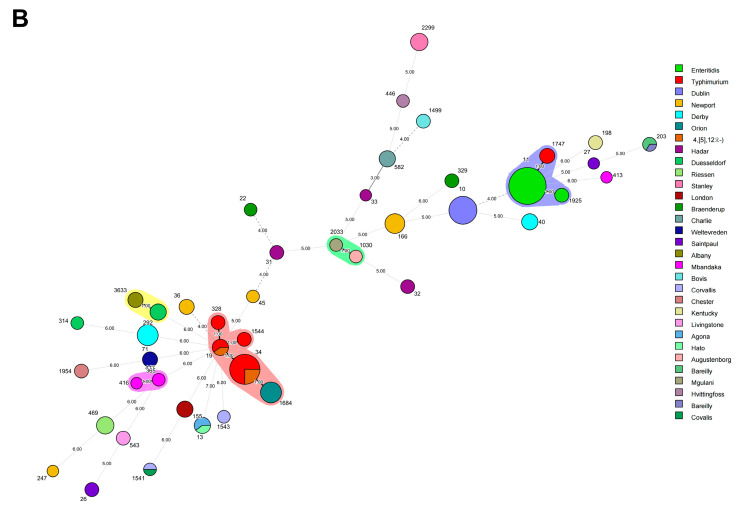
Minimum spanning tree for *Salmonella* on the Qinghai–Tibet Plateau. (**A**) Minimum evolution tree generated from regional characteristics. (**B**) Minimum evolution tree generated from serotypes as features.

Our analysis indicates that the allelic genetic diversity of the isolated strains ranges from 0.4963 to 0.9135 (0.4963 ≤ Ds ≤ 0.9135), with the *aroC* gene exhibiting the highest polymorphism ratio and the *purE* gene showing the lowest polymorphism ratio. In addition, based on the sequence types (STs) of *Salmonella* and seven different housekeeping genes, as well as the corresponding allelic profiles of the strains, the 223 strains of *Salmonella* were divided into 44 STs (Figure 7).

The most prevalent ST was ST11 (16.59%, 37/223), followed by ST34 (10.76%, 24/223), ST10 (8.52%, 19/223), ST71 (4.04%, 9/223), ST1684 (4.04%, 9/223), ST166 (3.59%, 8/223), and ST469(2.69%, 6/223). The proportions of ST13, ST19, ST40, ST155, ST292, and ST582 all corresponded to a rate of 4.04%, or 9/223. The STs ST36, ST365, ST1747, and ST363 contained four strains each (1.79%, 4/223). The STs ST26, ST31, ST32, ST198, ST203, ST328, ST329, ST543, ST1499, ST1544, ST1925, and ST1543 contained three strains each (1.35%, 3/223). The STs ST22, ST45, ST314, ST417, ST446, ST1030, ST1541, ST1543, and ST2033 contained two strains each (0.90%, 2/223). The STs ST27, ST33, ST247, ST413, and ST416 each contained only one strain (0.45%, 1/223) (Figure 7A).

ST11 had the most widespread distribution and was present in 10 different cities in Tibet and Qinghai. The main serotype of these strains was Enteritidis *Salmonella* (Lhasa, n = 5; Qamdo, n = 3; Nagqu, n = 7; Shigatse, n = 3; Shannan, n = 4; Ngari, n = 3; Xining, n = 7; Haibei, n = 1; Huangnan, n = 2; Yushu, n = 2). ST34 mainly originated in Tibet (Lhasa, n = 7; Nyingchi, n = 4; Qamdo, n = 2; Shigatse, n = 1; Shannan, n = 1), but some of the strains in this sequence type were also found in Qinghai (Huangnan, n = 6; Haixi, n = 3). ST19, ST27, ST32, ST33, ST155, ST203, ST543, ST1499, ST1747, ST1544, ST1954, and ST2033 were only detected in Tibet, while ST26, ST31, ST36, ST247, ST413, ST446, ST1030, ST1543, ST1925, and ST2299 were only detected in Qinghai (Figure 7B).

### 3.7. Phylogenetic and Genetic Diversity Analyses

The minimum spanning tree was mainly composed of five clonal complexes (CC1–CC5), accounting for 36.64% (104/223) of all the isolated strains. CC1 contained 44 strains (19.73%, 44/223), including ST19, ST34, ST328, ST1544, and ST1684; CC2 contained only four strains (1.79%, 4/223), including ST1030 and ST2033; CC3 contained 44 strains (19.73%, 44/223), including ST11, ST1747, and ST1925; and CC4 contained nine strains (4.04%, 9/223), including ST292 and ST3633, while CC5 contained only three strains (1.35%, 3/223), including ST416 and ST417. Based on the different alleles of the strains, the evolutionary relationships show that CC1, CC4, and CC5 are genetically close, belonging to the same evolutionary branch. CC2 has a distant evolutionary relationship with CC1, CC4, CC5, and CC3, while the relationship between CC3 and CC5 is the most distant. In summary, the isolates of the CCs are markedly clustered together in both the phylogenetic and minimum spanning trees. The systematic evolution tree reflects the evolutionary genetic differences between the different isolates. Isolates on the same evolutionary branch come from multiple regions. They are uniformly distributed in terms of their serotypes but are not correlated, indicating that there was cross-transmission and connection in the genetic evolution of *Salmonella* in yaks on the Qinghai–Tibet Plateau. The evolutionary relationships between ST26, ST203, ST247, and ST2299 and the other STs were the most distant within the STs. The tree generated is consistent. Isolates from the same branch come from different regions, while isolates from some regions (Haibei and Shannan) demonstrate relatively close evolution and are clustered on the same evolutionary branch.

In order to better understand the genetic diversity among the isolated strains, Simpson’s index (Ds) was used to determine genetic diversity. The total 223 isolated strains were divided into 44 STs, resulting in a Ds value of 0.9632. The genetic diversity in Tibet and Qinghai was similar. In Tibet, 127 strains were divided into 32 STs (Ds = 0.9521), while in Qinghai, 96 strains were divided into 29 STs (Ds = 0.9473). In the Tibet region, Nyingchi has the highest genetic diversity (19 isolated strains divided into 13 STs; Ds = 0.9221), followed by Qamdo (12 isolated strains divided into seven STs; Ds = 0.9142) and Shannan (16 isolated strains divided into eight STs; Ds = 0.5131). The genetic diversity in Shigatse is the lowest (24 strains divided into nine STs; Ds = 0.4294).

In the Qinghai region, Hainan has the highest genetic diversity (five strains divided into five STs, Ds = 0.9531), followed by Haibei (eight strains divided into six STs; Ds = 0.9326) and Haidong (eleven strains divided into eight STs; Ds = 0.9013). The lowest genetic diversity is in Guoluo (seven strains divided into three STs; Ds = 0.4490). Based on their richness of genetic diversity, the remaining regions were ranked as follows: Huangnan (Ds = 0.8525), Nagqu (Ds = 0.8251), Yushu (Ds = 0.7468), Lhasa (Ds = 0.6438), and Ngari (Ds = 0.5867).

**Figure 8:**
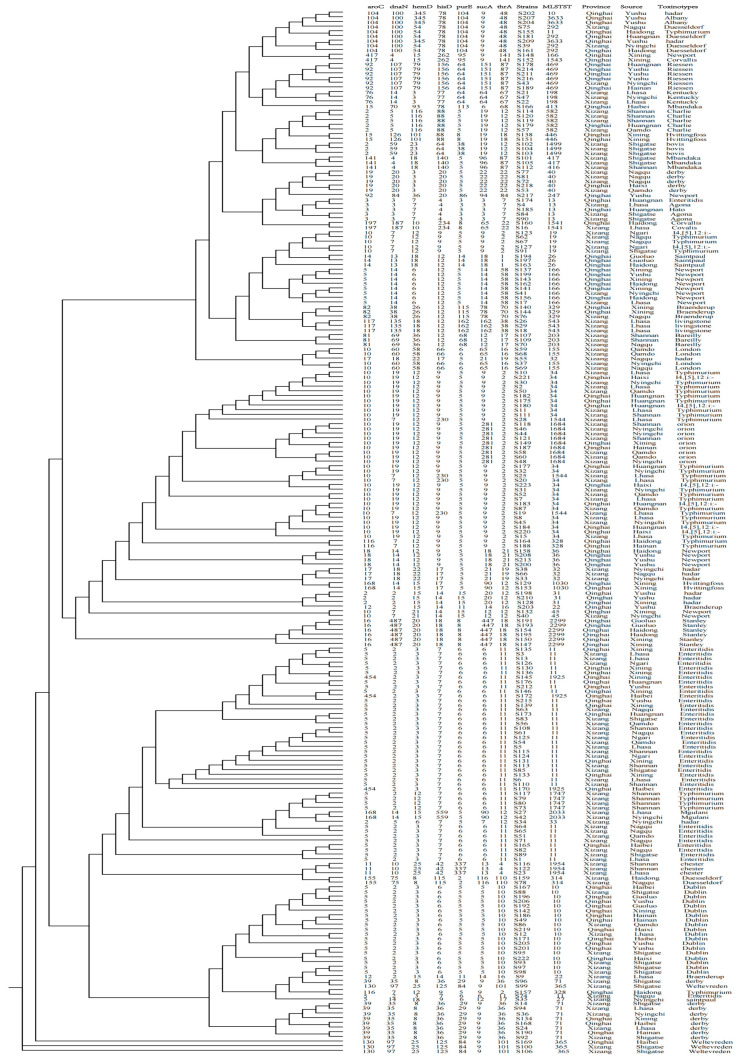
Phylogenetic tree and allelic profile of 223 strains of *Salmonella* isolated from yaks, along with serotype information.

The circles represent sequence types, the size of a circle represents the number of strains, and the numbers on the circles represent the names of the STs. The numbers on connecting lines between two circles indicate genetic evolutionary differences between two STs. Strains of the same color are part of the same clonal complex (CC) (Figure 8).

## 4. Discussion

Further investigation of *Salmonella*, a significant zoonotic bacterial pathogen [43], is necessary to comprehend its antimicrobial resistance, virulence factors, serotypes, and genetic origins. This knowledge could contribute to a deeper understanding of the prevalence of *Salmonella* in yaks on the Qinghai–Tibet Plateau and facilitate more effective strategies for preventing salmonellosis in these economically vital plateau animals. Our study reports a prevalence of 18.25% (223/1222) in the yak samples [44], which was lower than the 27% found in Dutch calf intestines, 41.8% in Spanish dairy cows, 45% in Wisconsin cattle farms, and 66.6% in Korean chicken meat. However, the prevalence is comparable to findings of 17.6% in Japanese pork and 12.9% in processed beef in Ethiopia; these rates suggest regional variations [45,46,47,48,49]. The prevalence of *Salmonella* also varies across livestock species, with differences observed in beef cattle, laying hens, and sheep [50,51,52]. The lower prevalence found in yaks (18.25%) compared to other regions may be attributed to the unique living conditions on the Qinghai–Tibet Plateau, which is characterized by high altitude, low temperature, low humidity, intense ultraviolet radiation, and limited bacterial carriers [53]. These conditions limit bacterial reproduction and transmission, aligning with the distinct microbial community structures and environmental factors found at high altitudes, where Gram-negative bacteria typically predominate over Gram-positive bacteria [54].

This study examined antimicrobial resistance in 223 *Salmonella* strains isolated from yak feces from the Qinghai–Tibet Plateau, focusing on resistance to 10 major antimicrobial classes [55]. The findings revealed a concerning prevalence of resistance to 26 antimicrobials, with the strains showing high rates of resistance to cephalexin (85.20%), penicillin (84.30%), ampicillin (82.96%), tetracycline (73.54%), and streptomycin (66.37%). Notably, *Salmonella* from fattening pigs in Hubei displayed a comparable resistance to tetracycline (93.46%), while retail chicken meat in Iran exhibited significant resistance to erythromycin (56.7%) [56,57]. Resistance to these antimicrobials, primarily first-generation β-lactams, may be attributed to their overuse in farming due to a lack of knowledge and expertise among farmers or herders. The strains were relatively sensitive to ciprofloxacin, sulfamethoxazole, cefotaxime, polymyxin B, vancomycin, and nalidixic acid, particularly Quinolones, which inhibit bacterial DNA synthesis. This study found that only two of the isolated strains were sensitive to ciprofloxacin and nalidixic acid, suggesting that while resistance to broad-spectrum antimicrobials is widespread, the emergence of super-resistant strains remains limited. Timely monitoring of drug susceptibility is crucial to mitigate resistance trends and effectively manage related diseases. The analysis of drug resistance genes in the 223 isolated strains highlighted their significant prevalence, including *bla_TEM_*, *bla_CTX_*, *ermB*, *rmtB*, *sul-2*, *tetB*, *armA*, *Mcr-2*, *bla_CMY_*, and *mcr-1*. This aligns with previous findings of the high prevalence of β-lactam resistance (*bla_CTX_* and *bla_TEM_*) in Spanish-pig-derived *Salmonella* [58] and the 85.7% prevalence of *bla_CMY_* in American-cattle-derived *Salmonella* [59]. Antimicrobial residues can disrupt bacterial ecosystems, fostering resistance. The coexistence of resistance phenotypes and gene carriage supports this notion [60], as substantiated by the high resistance of the *Salmonella* isolates to β-lactams (such as ampicillin and penicillin) and the prevalence of resistance genes.

Toxins, primarily proteins [61], are encoded by genes on bacterial chromosomes or mobile genetic elements like plasmids or transposons in pathogens [62]. These genes contribute to pathogenicity through mechanisms such as adhesion, invasion, and toxin production [63]. In this study, 15 virulence genes from nine categories (including Enterotoxin, SPI-1-5, Bacteriophage, Pili, and Toxic plasmids) were assessed in the 223 *Salmonella* isolates. The Enterotoxin gene *STN* had the highest detection rate (100%), followed by *orgA* (91.48%), while rates ranging from 70% to 40% were found for the other genes. A previous study in China [64] found widespread SPI-1 and SPI-2 carriage in isolates from the 2004–2019 period, while a Malaysian study [65] also reported high sopB carriage (99%). Our experiment detected sopB at a rate of 73.99%, which was slightly lower than the finding in Malaysia, but all strains carried *invA*, consistent with our results. Pathogenicity islands, acquired through horizontal gene transfer [66], contribute specific virulence traits, often accompanied by additional genetic elements outside these islands [67], which are hallmarks of pathogenicity.

Based on different combinations of H and O antigens, *Salmonella* can be classified into different serotypes [68]. In characterizing the serotypes of the 223 *Salmonella* isolates, this study uncovered 30 distinct serotypes. Enteritidis (40, 17.94%) was the most prevalent, followed by Typhimurium (31, 13.90%), aligning with findings in Korea, where Enteritidis accounted for 16.1% of human-derived strains [69]. The predominant serotypes of *Salmonella* strains isolated from human, chicken, and cattle populations in Iran were also Enteritidis (37%) and Typhimurium (35.3%), but with higher prevalences compared to the results of this study [70]. Enteritidis was also dominant (22.7%) in river samples from Georgia, suggesting a global trend of this serotype’s prevalence that is key to environmental and animal health [71]. In order to maintain a sustainable balance, continuous monitoring of *Salmonella* Enteritidis is crucial within the One Health framework.

The MLST analysis of the *Salmonella* strains identified 44 sequence types (STs) and five clonal complexes, with an average of 10.47 loci alleles. Xu L applied multi-locus sequence typing (MLST) to 52 clinical fecal *Salmonella* isolates from children with diarrhea in Guangdong, China, and found that the predominant serotypes were ST34 and ST19, with 10 clonal complexes (CCs); this is similar to the most prevalent ST types (ST11 and ST34) identified in this study but with a higher number of CCs [72]. The intestinal *Salmonella* isolated from live retail poultry in six provinces and cities in China formed four main clusters, with genetic diversity among the isolates (Ds = 0.9255) [73]. A higher diversity in ST clustering was found in Tibet and Qinghai, with a Simpson’s index of 0.9754.

The most common ST in the 223 isolates was ST11 (16.59%, 37/223), followed by ST34 (10.76%, 24/223). In Bangladesh, ST11, ST198, and ST214 predominate in poultry farms, and all are resistant to tetracyclines [74]. In this study, ST11 and ST198 showed high resistance to tetracyclines, while ST11 was dominant in *Salmonella* found in chickens in the central Malay Peninsula. The presence of *tetB* and *bla_TEM_* resistance genes in the ST11 strains suggests a correlation among phylogeny, antimicrobial resistance, and specific genetic traits, which could be further explored using additional methods [75]. This study has certain reference value for animal disease prevention and control policies in relevant cities in the Qinghai–Tibet Plateau area, and provides certain reference significance for the intervention measures of local farmers on yak *Salmonella* infection. At the same time, under the background of “One Health”, the situation of yak *Salmonella* infection in the Qinghai–Tibet Plateau area is closely related to global biosecurity, so the investigation of animal disease epidemiology has important significance for both the region and the world. In the follow-up studies, we will continue to track the dynamic epidemic changes of *Salmonella* and investigate other related diseases of yak, as well as conduct deeper exploration and mechanism research.

## 5. Conclusions

This pioneering study on yaks on the Qinghai–Tibet Plateau reports a high prevalence of *Salmonella* with broad antimicrobial resistance: 223 strains of *Salmonella* showed high resistance to antibiotics such as ampicillin (88.79%), penicillin (86.55%), erythromycin (81.17%), kanamycin (81.17%), polymyxin B (74.89%), etc. The detection rates of resistant genes accounting for the top five were as follows: *bla_TEM_* (69.51%), *bla_CTX_* (61.88%), *ermB* (58.30%), *rmtB* (46.19%), *sul-2* (40.36%). A total of 15 different types of virulence genes were detected, with *STN* (100%), *orgA* (91.48%), and *mgtC* (75.34%) having high detection rates. The MLST results showed that 45 STs were identified in the 223 strains of *Salmonella*, including five clonal complexes (CCs), with the main lineages being ST34, ST11, and ST19. The genetic diversity of the 223 strains was high, with a Simpson index of 0.9528. Further research is needed to uncover additional factors and strategies for combating antimicrobial resistance in yaks.

## Figures and Tables

**Table 1:** Isolation rates for *Salmonella* in different regions.

Province	Separation Date	Location of Isolation	Total Number of Samples	Isolation Rate
Tibet	2018/5	Lhasa	115	25.2% (29/115)
	2018/9	Nyingchi	120	15.8% (19/120)
	2018/10	Qamdo	60	20.0% (12/60)
	2019/7	Nagqu	100	22.0% (22/100)
	2020/10	Shigatse	104	23.1% (24/104)
	2021/11	Shannan	64	25.0% (16/64)
	2022/5	Ngari	22	22.7% (5/22)
	Total 1		585	21.7% (127/585)
	2018/6	Xining	147	17.7% (26/147)
	2018/6	Haidong	70	15.7% (11/70)
Qinghai	2019/8	Haibei	79	10.1% (8/79)
	2019/8	Huangnan	60	11.8% (13/60)
	2020/5	Hainan	33	21.7% (5/33)
	2020/5	Guoluo	43	16.3% (7/43)
	2021/9	Yushu	170	11.8% (20/170)
	2022/6	Haixi	35	17.1% (6/35)
	Total 2		637	15.1% (96/637)
	Total 3		1222	18.3% (223/1222)

**Table 2:** Housekeeping genes, fragment size, number of alleles, average number of alleles, and Simpson’s diversity index (Ds) for *Salmonella* strains from yaks.

Genes	*aroC*	*dnaN*	*hemD*	*hisD*	*purE*	*sucA*	*thrA*
Fragment size/bp	826	833	666	894	510	643	852
Number of alleles	32	26	22	24	15	27	25
Average number of alleles	17.24	11.63	9.28	17.33	3.40	8.31	6.11
Simpson’s diversity index (Ds)	0.9135	0.8767	0.7831	0.8236	0.4963	0.9024	0.8534

## Data Availability

The original contributions presented in this study are included in the article/Supplementary Materials, and further inquiries can be directed to the corresponding author.

## Supplementary Materials

The following supporting information can be downloaded at: https://www.mdpi.com/article/10.3390/ani14243697/s1, Data Sheet S1: Tables S1–S3: Specific information on the antibiotics used in the experiment, specific information on the primers used, and the specific antimicrobials resistance patterns of the isolated strains. Data Sheet S2: Specific Information and Origin of the Yak Dung Samples Collected, as Well as the Yak’s Condition.
