# Supplementary material for: An Epidemiological Study on Salmonella in Tibetan Yaks from the Qinghai–Tibet Plateau Area in China
Source: Animals (Basel). 2024 Dec 21;14(24):3697. doi: 10.3390/ani14243697 (PMC11672581; doi:10.3390/ani14243697)
Supplement: Supplementary file 1 [file animals-14-03697-s001.zip › Data Sheet S1.pdf]

Supplementary Table S1 Types of Antibacterial Drugs and Determination Criteria for Drug Resistance

Types of antibiotics	Drugs	The size of the zone of inhibition(mm)		
		Resistant	Intermediary	Sensitive
beta-lactam antibiotics	Cefotaxime	≤14	14-23	≥23
	Cefazolin	≤14	14-18	≥18
	Ampicillin	≤13	13-17	≥17
	Cefuroxime	≤14	14-23	≥23
	CFP	≤15	15-21	≥21
	Penicillin	≤13	13-17	≥17
	Cephalexin	≤14	14-18	≥18
	Oxacillin	≤10	10-13	≥13
Macrolide antibiotics	Azithromycin	≤13	13-18	≥18
	Erythromycin	≤13	13-23	≥23
Aminoglycoside antibiotics	Gentamicin	≤12	12-15	≥15
	Kanamycin	≤13	13-18	≥18
	Streptomycin	≤11	12-15	≥15
Tetracycline antibiotics	Minocycline	≤12	12-16	≥16
	Doxycycline	≤12	12-16	≥16
	Tetracycline	≤14	14-19	≥19
Lincosamide antibiotics	Lincomycin	≤10	10-20	≥20
Chloramphenicol antibiotics	Chloramphenicol	≤12	12-18	≥18
	Florfenicol	≤12	12-18	≥18
Polypeptide antibiotics	Vancomycin	≤9	9-12	≥12
	Bacitracin	≤8	8-13	≥13
	Polymyxin B	≤8	8-12	≥12
Quinolone antibiotics	Ciprofloxacin	≤15	15-21	≥21
	nalidixic acid	≤13	13-19	≥19
Nitrofuran antibiotics	Furazolidone	≤14	14-17	≥17
Sulfonamide antibiotics	Sulfamethoxazole	≤12	12-17	≥17

Supplementary Table S2 Salmonella identification primers, resistance genes, virulence genes, and housekeeping gene primers information

Gene name	Primer sequence（5′-3′）	Fragment size(bp)
InvA	F：GTGAATTATCGCACGTTCGGCAA<br>R：TCATCGCACGTCAAAGGAACC	284
tetA	F：GCTACATCCTGCTTGCCTTC<br>R：CATAGATCGCCGTGAAGAGG	210
tetB	F：TTGGTTAGGGGCAAGTTTTG<br>R：GTAATGGGCCAATAACACCG	659
armA	F：GGGTCTTACTATTCTGCCTAT<br>R：ATTCCCTTCTCCTTTCCAG	503
rmtB	F：TTTCTGCGGGCGATGTAA<br>R：AGTTCTGTTCCGATGGTCTTT	523
sul-1	F：GTGACGGTGTTCGGCATTCT<br>R：CCGAGAAGGTGATTGCGCT	780
sul-2	F：GGCTTATTTTCCTGCTAATTGG<br>R：GCAATGTGATCCATGATGTCGC	710
floR	F：GACGCCCGCTATGATCCAAC<br>R：GAGCATCGCCAGTATAGCCAA	406
gyrA	F：ACGTACTAGGCAATGACTGG<br>R：AGAGTCGCCGTCGATAGAAC	191
gyrB	F：GCGCTGTCCGAACTGTACCT<br>R：TGATCAGCGTCGCCACTTCC	169
bla_{TEM}	F：TCGCCGCATACACTATTCTCAGAATGA<br>R：ACGCTCACCGGCTCCAGATTT	445
bla_{CTX}	F：ATGTGCAGYACCAGTAARGTATGGC<br>R：TGGGTAATAGTACCAGAACAGCGG	593
bla_{CMY}	F：ACAGCCTCTTTCTCCACATT<br>R：ATTGCCTCTTCGTAACTCATT	545

bla$_{OXA}$	F：ACACAATACATATCAACTTCGC<br>R：AGTGTGTTTAGAATGGTGATC	814
bla$_{KPC}$	F：CGAACCATTCGCTAAACTCG<br>R：CCGATCCCTGCAATATGCTC	733
bla$_{LAP}$	F：CTCGCTGTACTTGTCATCCTTG<br>R：CACGATATTGATATTAGCCCG	749
bla$_{SHV}$	F：ATGCGTTATATTCGCCTGTG<br>R：TGCTTTGTTATTCGGGCCAA	747
ereA	F：GCCGGTGCTCATGAACTTGAG<br>R：CGACTCTATTCGATCAGAGGC	420
ermB	F：CGAGTGAAAAAGTACTCAACC<br>R：GCCGTGTTTCATTGCTTGATG	190
mcr-1	F：CGGTCAGTCCGTTTGTTC<br>R：CTTGGTCGGTCTGTAGGG	309
Mcr-2	F：TTGGCACTGTATTTTGCATTT<br>R：TTAACGAAATTGGCTGGAACA	542
Stn	F：CTTTGGTCGTAAAATAAGGCG<br>R：TGCCCAAAGCAGAGAGATTC	260
orgA	F：TTTTTGGCATGCATCAGGGAACA<br>R：GGCGAAAGCGGGGACGGTATT	255
sopE	F：ATTGTTGGCGTTGGCATCGT<br>R：AATGCGAGTAAAGATCCGGCCT	386
avrA	F：CCTGTTTGAGCGTCTGG<br>R：AGAAGAGTTCGTTGAATGTCC	422
ttrB	F：ATGTGGACGGAGTCAATATGG<br>R：GTGGCGATGCGGCTATGG	608
sseL	F：TTCCGCGACACCGACCTTTCTAA<br>R：TTCTTGAACCGACCTTGCGTTGC	169
rhuM	F：GCCAGCTTATAGTGCCAAGC	202

	R：ATAATCACGGTTCCGCGTAG	
mgtC	F：TGACTATCAAGCTCCAGTGAAT<br>R：ATTTACTGGCCGCTATGCTGTTG	655
orfL	F：GCCCCGCCTGAGCCTGTGTTGC<br>R：AGGTCGGCTGTTGAGTTGGATA	340
sopB	F：CGGACGCAGCCAAAAAGAAGA<br>R：TAGTGATGGTATGCTGGTGTATT	220
pipD	F：CGGCGATCATGACTTTGAT<br>R：CGTTATCTTCGGATCGTAA	405
sodC1	F：GTTCAGCAAGGCAGAGAATAC<br>R：CCAGTGGACAGGTTTATCG	444
lpfC	F：GCCCCGCCTGAGCCTGTGTTGC<br>R：AGGTCGCGCTGTTGATTGGATA	641
spvC	F：ACTCCTTGCACACCAATGCGGA<br>R：TGTCTCTGCATTCGCCACCATCA	571
pefA	F：CCTGTGACCTACCACTTCTG<br>R：TTGTAAGCCACGCGAAAGATG	418
aroC	F：CCTGGCACCTCGCGCTATAC<br>R：CCACACACGGATCGTGGCG	826
dnaN	F：ATGAAATTTACCGTTGAACGTGA<br>R：AATTTCTCATTCGAGAGGATTGC	833
hemD	F：GAAGCGTTAGTGAGCCGTCTGCG<br>R：ATCAGCGACCTTAATATCTTGCCA	666
hisD	F：GAAACGTTCCATTCCGCGCAGAC<br>R：CTGAACGGTCATCCGTTTCTG	894
purE	F：ATGTCTTCCCGCAATAATCC<br>R：TCATAGCGTCCCCCGCGGATC	510
sucA	F：AGCACCGAAGAGAAACGCTG<br>R：GGTTGTTGATAACGATACGTAC	643

thrA	F：GTCACGGTGATCGATCCGGT<br>R：CACGATATTGATATTAGCCCG	852

Supplementary Table S3 Drug resistance patterns of Salmonella yak in Qinghai and Tibet.

Patterns of drug resistance	No. (%) of drug resistant isolates		
	Tibet(n=127)	Qinghai(n=96)	Total(n=223)
**Single**	**0**	**0**	**0**
**Double**	**2(1.57)**	**1(0.45)**	**3(1.35)**
BET-AGA	1(50.00)	0(0.00)	1(33.3)
BET-TET	1(50.00)	1(100.00)	2(66.7)
**Triple**	**23(18.11)**	**14(14.58)**	**37(16.59)**
BET-AGA-TET	12(52.17)	7(50.0)	19(51.35)
BET-AGA-CAP	5(21.74)	3(21.43)	8(21.62)
BET-AGA-MAC	2(8.70)	1(7.14)	3(8.11)
BET-TET-MAC	3(13.04)	0(0.00)	3(8.11)
BET-AGA-POL	1(4.35)	2(14.29)	3(8.11)
BET-TET-POL	0(0.00)	1(7.14)	1(2.70)
**Quadruple**	**33(25.98)**	**32(33.33)**	**65(29.15)**
BET-AGA-TET-CAP	12(36.36)	11(34.38)	28(43.08)
BET-AGA-TET-MAC	10(30.30)	7(21.88)	20(30.77)
BET-AGA-TET-POL	2(6.06)	9(28.13)	12(18.46)
BET-AGA-CAP-MAC	1(3.03)	0(0.00)	1(1.54)
BET-AGA-CAP-POL	1(3.03)	1(3.13)	2(3.08)
AGA-TET-CAP-MAC	2(6.06)	3(9.38)	6(9.23)
AGA-TET-CAP-POL	3(9.09)	0(0.00)	3(4.62)
TET-CAP-MAC-POL	2(6.06)	1(3.13)	3(4.62)
**Quintuple**	**34(26.77)**	**25(26.04)**	**59(26.46)**
BET-AGA-TET-CAP-MAC	12(35.29)	6(24.00)	18(30.51)
BET-AGA-TET-CAP-POL	9(26.47)	7(28.00)	16(27.12)
BET-AGA-TET-CAP-LIN	5(14.71)	2(8.00)	7(11.86)
BET-AGA-TET-CAP-FZ	2(5.88)	1(4.00)	3(11.86)

AGA-TET-CAP-MAC-POL	2(5.88)	0(0.00)	2(3.34)
AGA-TET-CAP-MAC-LIN	1(2.94)	2(8.00)	3(5.08)
AGA-TET-CAP-POL-LIN	1(2.94)	3(12.00)	4(6.78)
TET-CAP-MAC-POL-LIN	2(5.88)	4(16.00)	6(10.17)
**Sextuple**	**17(13.39)**	**12(12.50)**	**29(13.00)**
BET-AGA-TET- CAP-MAC-POL	7(41.18)	7(58.33)	14(48.28)
BET-AGA-TET-CAP-MAC-LIN	3(17.65)	0(0.00)	3(10.34)
BET-AGA-TET-CAP-MAC-FZ	3(17.65)	2(16.67)	5(17.24)
AGA-TET-CAP-MAC-POL-LIN	1(5.88)	2(16.67)	3(10.34)
AGA-TET-CAP-MAC-POL-FZ	1(5.88)	1(8.33)	2(6.90)
TET-CAP-MAC-POL-LIN-FZ	2(11.76)	0(0.00)	2(6.90)
**Septuple**	**10(7.87)**	**9(9.38)**	**19(8.52)**
BET-AGA-TET-CAP-MAC-POL-LIN	3(30.00)	333.33)	6(31.58)
BET-AGA-TET-CAP-MAC-POL-FZ	3(30.00)	3(33.33)	6(31.58)
AGA-TET-CAP-MAC-POL-LIN-FZ	1(10.00)	1(11.11)	2(10.53)
BET-AGA-TET-CAP-MAC-POL-LIN-QUIN	3(30.00)	2(22.22)	5(26.32)
**Octuple**	**2(1.57)**	**2(2.08)**	**4(1.79)**
BET-AGA-TET-CAP-MAC-POL-LIN-FZ	1(50.00)	0(0.00)	1(25.00)
BET-AGA-TET-CAP-MAC-POL-LIN-Sas	1(50.00)	2(100.00)	3(75.00)
**Nonuple**	**4(3.15)**	**1(1.04)**	**5(2.24)**
BET-AGA-TET-CAP-MAC-POL-LIN-FZ- Sas	1(50.00)	0(0.00)	1(20.00)
BET-AGA-TET-CAP-MAC-POL-LIN-FZ-QUIN	3(75.00)	1(100.00)	4(80.00)
**Decuple**	**2(1.57)**	**0(0)**	**2(0.90)**
BET-AGA-TET-CAP-MAC-POL-LIN-FZ-Sas-QUIN	2(100.00)	0(0.00)	2(100.00)

Notes:

Notes: PEN, Penicillin antibiotics

CEP, Cephalosporin antibiotics

MAC, Macrolide antibiotics

AGA, Aminoglycoside antibiotics

TET, Tetracycline antibiotics

LIN, lincosamide antibiotics

CAP, Chloramphenicol antibiotics

POL, Polypeptide antibiotics

QUIN, Quinolone antibiotics

FZ, Furazolidone antibiotics

SAs, Sulfonamide antibiotics

BET， Beta-lactam antibiotics
